# ITS2 rRNA Gene Sequence–Structure Phylogeny of the Chytridiomycota (Opisthokonta, Fungi)

**DOI:** 10.3390/biology14010036

**Published:** 2025-01-05

**Authors:** Yuveantheni Kalaiventhan, Kensuke Seto, Matthias Wolf

**Affiliations:** 1 Department of Bioinformatics, Biocenter, University of Würzburg, Am Hubland, 97074 Würzburg, Germany; yuveantheni.kalaiventhan@stud-mail.uni-wuerzburg.de; 2 Faculty of Environment and Information Sciences, Yokohama National University, 79-7 Tokiwadai, Hodogaya, Yokohama, Kanagawa 240-8501, Japan; kensuke.seto0427@gmail.com

**Keywords:** Chytridiomycota, internal transcribed spacer 2, ITS2, phylogeny, secondary structure, sequence-structure, rRNA

## Abstract

To date, standard rRNA marker genes have had limited success in resolving the phylogeny of the phylum Chytridiomycota. Whereas the conserved and easily alignable ribosomal small subunit 18S rRNA gene had problems resolving nodes relating orders, the internal transcribed spacer 2 (ITS2) has been claimed to not be alignable for this group of organisms. Although the ITS2 is a fast-evolving locus, its secondary structure is well conserved. To improve the accuracy and robustness of Chytridiomycota phylogeny, in this study, we attempt, for the first, time to reconstruct an ITS2 sequence–structure phylogeny using the primary sequence and the secondary structure information simultaneously in inferring alignments and trees. Although currently only possible for a fraction of the available data, we show a well-supported ITS2 tree for selected organisms. The results are promising for further exploration of the large number of available ITS2 sequences.

## Simple Summary

The phylogeny of the Chytridiomycota, a group of fungi, could not be well resolved with standard rRNA marker genes. Typically, the primary sequence information was used in sequence comparisons and in inferring relationships between sequences. However, including RNA secondary structure information improves accuracy and robustness in reconstructions of phylogenetic trees. Therefore, in this study, we reconstruct an ITS2 rRNA gene sequence–structure phylogeny using the RNA primary sequence and the RNA secondary structure information simultaneously in inferring alignments and trees. We show a well-supported ITS2 sequence–structure tree for selected organisms of the Chytridiomycota.

## 1. Introduction

The phylum Chytridiomycota included all posteriorly uniflagellated zoosporic osmotrophs until multigene-based phylogenies revealed that the Chytridiomycota phylum was paraphyletic. Today three monophyletic phyla of zoosporic osomotrophic opisthokonts, namely, Chytridiomycota, Neocallimastigomycota, and Blastocladiomycota, are distinguished [1,2,3,4,5,6,7,8]. Chytridiomycota currently contains 13 orders according to Wijayawardene et al. [9], with no consideration given to the recently proposed Zygorhizidiales and Zygophlyctidales [10]. The current taxonomy is based on multi-marker analyses [4,11,12].

The internal transcribed spacer 2 (ITS2) of the nuclear ribosomal repeat unit is one of the most applied markers in phylogenetics and barcoding [13]. It is a fast-evolving locus, which makes it appropriate for studies at low taxonomic ranks (i.e., species and genera), while its secondary structure is well preserved such that phylogenetic tree reconstructions are also possible at higher taxonomic ranks [14]. In addition, it is established that including RNA secondary structure information generally improves accuracy and robustness in reconstructions of phylogenetic trees [15]. A “how to” on simply aligning rRNA sequences and their individual secondary structures simultaneously via an automatic approach has been reviewed and discussed by Wolf et al. [16].

To date, standard rRNA marker genes have had limited success in resolving the phylogeny of the Chytridiomycota. Whereas the conserved and easily alignable ribosomal small subunit 18S rRNA gene had problems resolving nodes relating orders [4], the ITS2 (although more than 5000 sequences are currently available) was claimed not to be alignable for this group of organisms [11].

In this study, we attempt, for the first time, to reconstruct an ITS2 sequence–structure phylogeny for the Chytridiomycota. Instead of using only the primary sequence information, and instead of guiding our sequence alignment on a consensus structure, here, we use the primary sequence and the secondary structure information simultaneously—merged and encoded in a new alphabet—to infer the alignments and trees. Our results are discussed in comparison to those of the available literature.

## 2. Materials and Methods

Methods employed include taxon sampling, automatic and manually optimized secondary structure prediction, sequence-only and sequence–structure alignment, and phylogenetic tree reconstruction.

**Figure 1:**
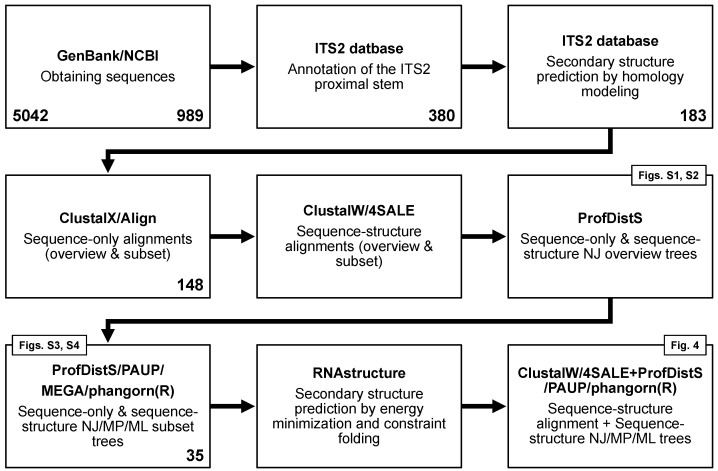
Flowchart of the analyses and filter steps performed. Numbers of sequences used in each step are additionally provided.

**Figure 2:**
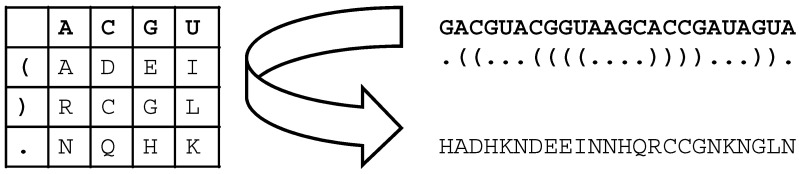
Using a 12-letter translation table, each of the four nucleotides, along with its structural pairing state (paired left, paired right, and unpaired), is translated into 1 of 12 letters. When generating an alignment, 4SALE reads this 1-letter-encoded information as protein sequences but uses a 12 × 12 (instead of a 20 × 20) scoring matrix with re-estimated substitution and gap costs. For the maximum parsimony and maximum likelihood analyses, PAUP* and phangorn/R directly use 1-letter-encoded .fasta files. Estimated from the data, maximum likelihood analysis of a 12 × 12 substitution model was used. ProfDistS (for neighbor-joining analyses) uses sequence–structure .xfasta files (including the bracket dot bracket notation), converting them internally to 1-letter-encoded .fasta files.

The methods used in this study are depicted in a flowchart (Figure 1). Chytridiomycota Hibbett et al. 2007 ITS2 rRNA gene sequences were obtained from GenBank (NCBI) [17,18] (retrieval date: 8 June 2024). Respective search strings are provided in the Supplementary Materials (Table S1). ITS2 sequences were annotated using hidden Markov models (HMMs) [14,19] implemented in the ITS2 database [20]. Sequence-only data were aligned with ClustalX 2.1 [21]. Encoded (see below) sequence–structure data were simultaneously aligned with ClustalW 1.83 [21], as implemented in 4SALE 1.7.1 [22,23]. Secondary structures were first predicted via homology modeling [24]. To obtain an overall secondary structure transfer percentage, each structure was predicted as one helix with several side arms (typically four) instead of an open structure with several helices; i.e., predicted structures include the proximal stem consisting of the last/first 25 nucleotides of the 5.8S/25S (5.8S/28S) interaction. Twenty-three template structures for homology modeling were obtained from the ITS2 database V [20] (cf. Table S1). The follow up phylogenetic sequence–structure approach is detailed in Keller et al. and Wolf et al. [15,16]. In a nutshell, we encode each nucleotide in three structural states (unpaired, paired left, paired right); i.e., we have used 12 × 12 scoring matrices for sequence–structure alignments, as well as 12 × 12 substitution models for likelihood-based sequence–structure tree reconstructions. This contrasts with the 4 × 4 matrices and models used in sequence-only analyses. Sequence-only alignments were edited using ALIGN 08 [25]. Sequence–structure alignments were edited using 4SALE. The final alignments are available from the authors on reasonable request. Taxa used or discarded are listed in Table S1. Reasons and/or filters (i.e., respective search strings) for sorting out sequences are specified there. More than 5000 sequences were reduced to 35 sequences/sequence–structure pairs by the various filters. In short, we only used sequences that are specified down to the species name, can be annotated by the full proximal stem, and have at least 50% structural homology. Phylogenetic trees were reconstructed using neighbor-joining (NJ) [26], maximum parsimony (MP) [27], and maximum likelihood (ML) [28] approaches. For sequence-only alignments, trees were reconstructed using default settings in ProfDistS 0.9.9 (NJ) [29], PAUP 4.0a (MP) [30], and MEGA 11 (ML) [31]. For sequence–structure alignments, trees were reconstructed using ProfDistS (NJ), PAUP (MP), and phangorn 2.12.1.1 (ML) [32]; the latter as implemented in R 4.3.0 [33]. File formats and sequence–structure encoding are explained in detail in Figure 2.

**Figure 3:**
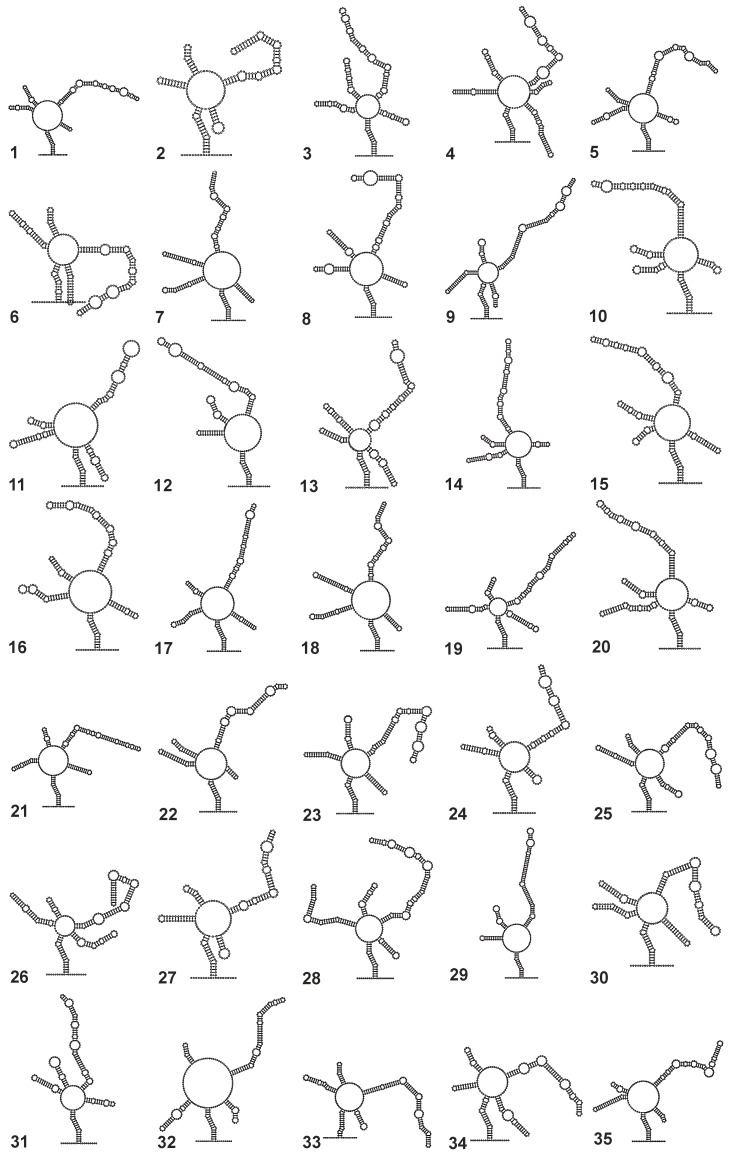
Individual ITS2 secondary structures for all 35 sequences from the subset. Using RNAstructure, structures were obtained through energy minimization and constraint folding. Constraints (lowercase letters/nucleotides forbidden to pair in RNAstructure) were applied to the central ring of the ITS2 in order to determine the optimal homologous helices. All chytridiomycote ITS2 secondary structures fit the common core structure known for eukaryotes; i.e., they consist of four helices, the third being the longest. Only *G. pollinis-pini* shows a fifth helix (with helices II a and b), and the two outgroup taxa do not show a fourth helix. 1: *H. littoreus*; 2: *T. subangulosum*; 3: *P. hirtus*; 4: *G. pollinis-pini*; 5: *P. uniporus*; 6: *R. carpophilum*; 7: *L. poculatus* (EU352770); 8: *C. vesicula*; 9: *R. globosum*; 10: *P. arcticum (T. arcticum)*; 11: *S. lactosolyticus* (AF216770); 12: *N. cameroonii*; 13: *G. semiglobifer*; 14: *F. jonesii*; 15: *R. rosea* (MT730933); 16: *R. rosea* (AF216765); 17: *S. lactosolyticus* (MF576127); 18: *L. poculatus* (MT595869); 19: *T. humboldtii*; 20: *B. kilaueaense*; 21: *S. dolichospermus*; 22: *U. aestuarii*; 23: *O. laminatus*; 24: *A. chaetifer*; 25: *P. lateralis*; 26: *A. argentinensis*; 27: *B. macroporosus*; 28: *Q. haematococci*; 29: *A. robustus*; 30: *P. pingflumenensis*; 31: *G. tanneri*; 32: *K. dichotomus*; 33: *A. chlorogonii*; 34: *D. gilberthii*; 35: *P. mangrovei*.

**Figure 4:**
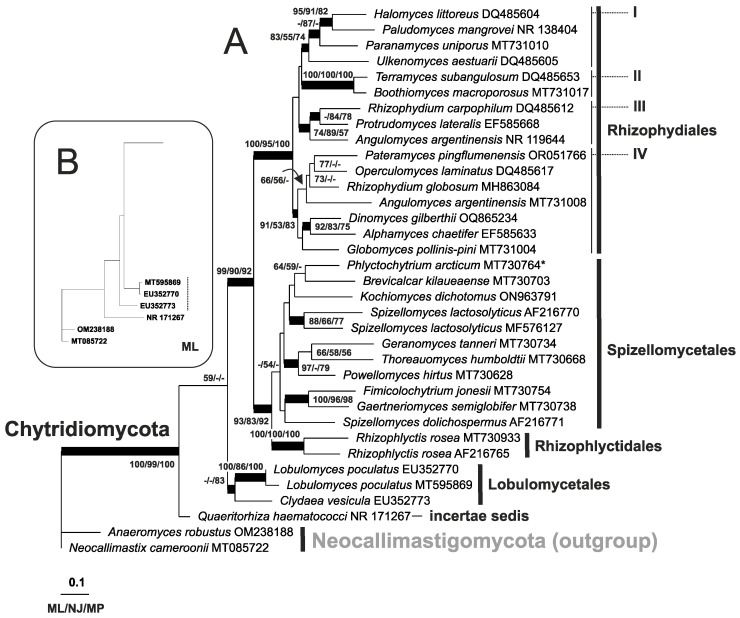
(**A**) ITS2 rDNA sequence–structure NJ subset tree (secondary structures were obtained through energy minimization and constraint folding). The tree was generated by using ProfDistS. Taxa names are accompanied by the GenBank accession number. Bootstrap values (>50), mapped at internal nodes, are from ML, NJ, and MP analyses. The MP tree was generated using PAUP*. The ML tree was generated using phangorn, as implemented in R. Topological differences are indicated by dashes and/or highlighted in (**B**). Trees were rooted with Neocallimastigomycota. The scale bar indicates evolutionary distances. * *P. arcticum* is synonym to *Triparticalcar arcticum*.

For all maximum likelihood analyses, substitution models were estimated from the data. For all trees, bootstrap support [34] was estimated (due to the complexity of the sequence–structure approach) based on only 100 pseudo-replicates. All trees were rooted with sequences/sequence–structure pairs from Neocallimastigomycota Hibbett et al. 2007. Prior to the 35 taxa subset trees, and in approaching these, for about 150 ITS2 sequences/sequence–structure pairs, large scale overview phylogenies (without a bootstrap analysis) were reconstructed via ProfDistS using only NJ. Taxa used for the subset trees are highlighted in the overview trees. The two overview trees (sequence-only and sequence–structure obtained via NJ) and the six subset trees, i.e., their bootstrap values (sequence-only and sequence–structure obtained via NJ, MP, and ML), are available in the Supplementary Material (Figures S1–S4). Starting from scratch, using RNAstructure 5.4 [35], for all 35 subset taxa, secondary structures were finally individualized through energy minimization and constrained folding (Figure 3). Constraints (lowercase letters/nucleotides forbidden to pair in RNAstructure) were applied to the central ring of the ITS2 secondary structure in order to deduce optimal homologous helices. ITS2 sequence–structure trees were again—and now finally—reconstructed on the optimized data (Figure 4). All sequence-only/sequence–structure trees, i.e., two overview trees (obtained via NJ) and nine subset trees (obtained via NJ, MP, or ML), are discussed in relation to each other and to the available literature.

## 3. Results

Figure S1 shows the relationships (as obtained via NJ) between 148 ITS2 sequence–structure pairs from organisms currently classified within Chytridiomycota. Several orders (Chytridiales, Cladochytriales, Polychytriales, Zygophlyctidales, Zygorhizidiales, Nephridiophagales, Polyphagales, Saccopodiales, Gromochytriales, Mesochytriales and Synchytriales) are absent as their sequences could not be obtained through NCBI or else they were filtered out through taxon sampling. Three orders, Spizellomycetales, Rhizophlyctidales, Rhizophydiales, as well as a group, *incertae sedis*, consisting of two strains of *Quaeritorhiza haematococci*, are monophyletic. Lobulomycetales are non-monophyletic, represented by four distinct clades. In the sequence-only NJ overview tree (Figure S2), Lobulomycetales are monophyletic. The genus *Quaeritorhiza* was identified as a sister taxon to Spizellomycetales in the sequence–structure NJ overview tree, whereas the sequence-only NJ overview tree shows *Quaeritorhiza* as a sister taxon to a large clade consisting of Rhizophydiales, Rhizophlyctidales, and a non-monophyletic “Spizellomycetales”. Rhizophlyctidales in the sequence–structure tree are sisters to *Q. haematococci* and Spizellomycetales, whereas in the sequence-only tree, they are only sisters to a “Spizellomycetales” subgroup (Figure S2). Backbone topologies and further sister group relations are discussed with the subset trees (see below, as well as Figures S3 and S4, and Figure 4).

The Chytridiomycota within the sequence–structure subset trees (ML/NJ/MP, using structures obtained via homology modeling, Figure S3) are highly supported against the outgroup (Neocallimastigomycota). The Chytridiomycota splits into Lobomycetales and all other chytridiomycote taxa. The clade containing all other Chytridiomycota then splits into *Globomyces pollinis-pini* (Rhizophydiales II) and the remaining taxa. The latter splits into Rhizophydiales I, with all other Rhizophydiales, expect for *G. pollinis-pini*, and a clade consisting of Rhizophlyctidales (two strains of *Rhizophylctis rosea*), *Quaeritorhiza haematococci* (currently classified *incertae sedis*), and Spizellomycetales. Further sister group relations are additionally supported: Within Lobomycetales, *Lobulomyces poculatus* (two strains) are sisters to *Chydaea vesicula*. *Quaeritorhiza haematococci* are sisters to Spizellomycetales. Within the latter, a larger group (*Gaertneriomyces semiglobifer* + *Spizellomyces dolichospermus*) + (*Fimicholochytrium jonesii* + *Spizellomyces lactosolyticus* (MF576127)) are sisters to the remaining Spizellomycetales. The latter split into *Kochiomyces dichotomus* + *Thoreauomyces humboldtii* and an additional five taxa clade. Therein, *Phlyctochytrium arcticum* (currently classified as *Triparticalcar arcticum* [36]) + *Geranomyces tanneri* are sisters to a clade consisting of *Spizellomyces lactosolyticus* AF216770 + *Powellomyces hirtus* and *Brevicalcar kilaueaense*. For detailed bootstrap values, see Figure S3. Within Rhizophydiales, *Rhizophydium globosom* + *Operculomyces laminatus* are sisters to *Dinomyces gilberthii*. Further supported sister groups are *Boothiomyces macroporosus* + *Terramyces subangulosum*, *Alphamyces chaetifer* + *Pateramyces pingflumenensis*, *Paludomyces mangrovei* + *Halomyces littoreus*, *Paranamyces uniporus* + *Angulomyces argentinensis* and *Rhizophydium carpophilum* + *Protrudomyces lateralis*. For detailed bootstrap values, see Figure S3.

In the sequence-only subset trees (Figure S4), the phylogenetic positions of Rhizophlyctidales and *Q. haematococci* exhibited a switch, as did some genera within other orders. A comparison of the bootstrap values within Spizellomycetales reveals slight differences between the sequence-only trees and the sequence–structure trees. Generally, more nodes are supported within the sequence–structure trees. The Rhizophydiales branch shows an equally low-to-non-supported backbone in all trees. In the sequence–structure tree, *P. uniporus* forms a moderately supported clade with *A. argentinensis*. However, in the sequence-only tree, *P. uniporus* forms a strongly supported clade with *P. mangrovei* and *H. littoreus*. For detailed bootstrap values, see Figure S4.

In the final sequence–structure subset trees (ML/NJ/MP, using optimized structures obtained through energy minimization and constraint folding), the general tree topology is as follows (Figure 4): Chythridiomycota are highly supported against the outgroup. *Q. haematococci* are sisters to all other chytridiomycote taxa, while the latter split into Lobulomycetales and the remaining Chytridiomycota. The next split defines two clades: the Rhizophydiales and a clade consisting of Rhizophlyctidales + Spizellomycetales. Additionally, within Rhizophydiales, four subclades (I–IV) can be distinguished (cf. Figure 4). In the following, the final tree is discussed in comparison to the literature and our supplementary trees described above (Figures S1–S4).

## 4. Discussion

Our different approaches to tree calculation show largely similar trees, which is in accordance with trees from the literature [4,11]. Nevertheless, among all our trees, and compared to other RNA-based trees from the literature [4,11], our final tree based on sequences and their optimized individual structures provides the best support. However, the known trees from the literature are only comparable to a certain extent because they were never calculated on ITS2 alone but always on a combination of different marker genes (18S + 5.8S + 28S) [4,11,12]. Although our approach is promising and allows us to reconstruct a robust tree using ITS2 alone, it is important to note that ITS2 data for several orders of the Chytridiomycota are currently not available and that we had to exclude a lot of existing data from our analysis for different reasons. For example, structural templates for homology modeling are currently available only for Lobomycetales, Rhizophydiales, Rhizophlyctidales, and Spizellomycetales; no data are available for Polychytriales, Nephridiophagales, Polyphagales, Saccopodiales, Gromochytriales, and Mesochytriales. A few ITS2 sequences were present for Cladochytriales, Zygophlyctidales, and Zygorhizidiales but could not be robustly annotated. More data were available for Synchytriales and Chytridiales, but they also could often not be robustly annotated and/or they showed less than 50% structural homology during homology modeling. Therefore, first, for these two latter groups, it would be worthwhile to look for better individual structures and/or better template structures for homology modeling. However, for all groups, there is certainly great potential for future analyses in predicting further template structures and/or in laboriously predicting every single optimal individual structure (typically energy-based with additional experience-based post-processing). In summary, the currently available annotatable and processable ITS2 data provide the following enkaptic tree structure for the Chytridiomycota: (Quaeritorhiza, (Lobomycetales, (Rhizophydiales, (Rhizophlyctidales, Spizellomycetales)))).

## 5. Conclusions

All the ITS2 secondary structures of chytridiomycetes used in this study correspond to the four-helix core structure known for eukaryotes, with the third helix being the longest. In contrast to sequence-only analyses, sequence–structure analyses yielded better-supported trees that generally matched the trees obtained through multi-marker analyses. Most of the sequences available in GenBank/NCBI are from unspecified species and/or are difficult to annotate, especially since sequencing often did not extend far enough into the flanking region of the 28S rDNA. However, our results are promising for further exploration of the large number of available ITS2 sequences.

## Data Availability

The GenBank numbers used are provided in the Supplementary Material. Alignments are available from the authors on reasonable request.

## Supplementary Materials

The following supporting information can be downloaded at https://www.mdpi.com/article/10.3390/biology14010036/s1: Figure S1: ITS2 rDNA sequence–structure NJ overview tree; Figure S2: ITS2 rDNA sequence-only NJ overview tree; Figure S3: ITS2 rDNA sequence–structure ML subset tree (using homology modeled ITS2 secondary structures); Figure S4: ITS2 sequence-only ML subset tree; Table S1: List of used taxa and GenBank numbers.
