# Supplementary material for: ITS2 rRNA Gene Sequence–Structure Phylogeny of the Chytridiomycota (Opisthokonta, Fungi)
Source: Biology (Basel). 2025 Jan 5;14(1):36. doi: 10.3390/biology14010036 (PMC11762872; doi:10.3390/biology14010036)
Supplement: Supplementary file 1 [file biology-14-00036-s001.zip › biology-3362839-supplementary.pdf]

Supplementary Materials

Supplementary Figure Legends

Figure S1. ITS2 rDNA sequence-structure NJ overview tree (secondary structures were obtained by homology modeling). The tree was generated by using ProfDistS. Taxa names are accompanied by the GenBank accession number. The tree is rooted with Neocallimastigomycota. The scale bar indicates evolutionary distances.

Figure S2. ITS2 rDNA sequence-only NJ overview tree. The tree was generated by using ProfDistS. Taxa names are accompanied by the GenBank accession number. The tree is rooted with Neocallimastigomycota. The scale bar indicates evolutionary distances.

Figure S3. ITS2 rDNA sequence-structure ML/MP/NJ subset trees (secondary structures were obtained by homology modeling). Taxa names are accompanied by the GenBank accession number. Bootstrap values (>50) mapped at internal nodes are from ML, NJ, and MP analyses. The ML tree was generated by using phangorn as implemented in R. The NJ tree was generated by using ProfDistS. The MP tree was generated by using PAUP*. Trees were rooted with Neocallimastigomycota. The scale bar indicates evolutionary distances. * *P. arcticum* is synonym to *Triparticalcar arcticum*.

Figure S4. ITS2 rDNA sequence-only ML/MP/NJ subset trees. Taxa names are accompanied by the GenBank accession number. Bootstrap values (>50) mapped at internal nodes are from ML, NJ, and MP analyses. The ML tree was generated by using MEGA. The NJ tree was generated by using ProfDistS. The MP tree was generated by using PAUP*. Trees were rooted with Neocallimastigomycota. The scale bar indicates evolutionary distances. * *P. arcticum* is synonym to *Triparticalcar arcticum*.

Supplementary Table Legends

Table S1. List of used taxa and GenBank numbers.

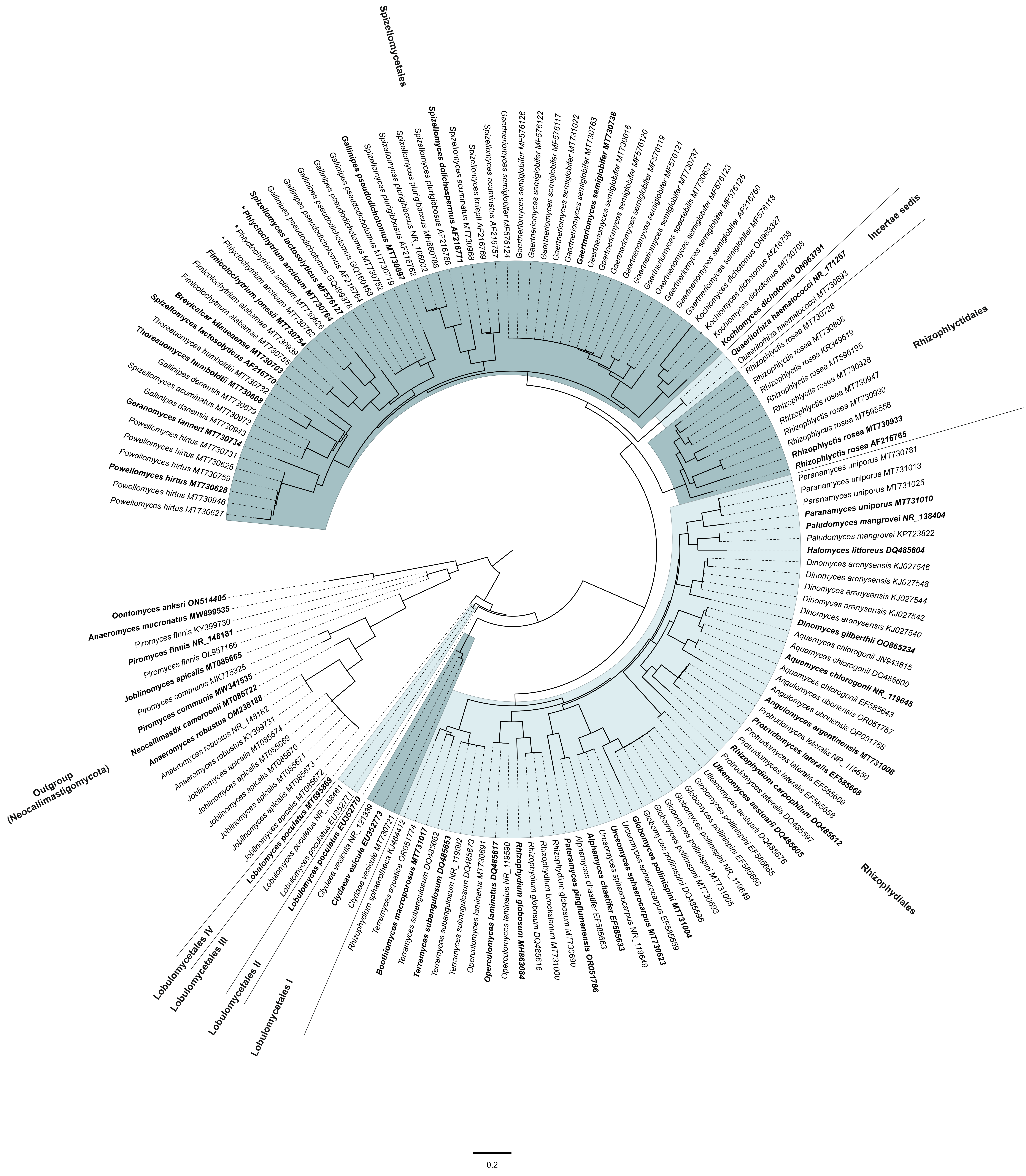

Spizellomycetales
Incertae sedis
Rhizophlyctidales
Rhizophydiales
Lobulomycetales I
Lobulomycetales II
Lobulomycetales III
Lobulomycetales IV
Outgroup
(Neocallimastigomycota)
Powellomyces hirtus MT730627
Powellomyces hirtus MT730946
Powellomyces hirtus MT730628
Powellomyces hirtus MT730759
Powellomyces hirtus MT730625
Powellomyces hirtus MT730731
Geranomyces tanneri MT730734
Gallinipes danensis MT730943
Spizellomyces acuminatus MT730972
Gallinipes danensis MT730679
Thoreauomyces humboldtii MT730668
Thoreauomyces humboldtii MT730732
Spizellomyces lactosolyticus AF216770
Brevicalcar kilaueaense MT730703
Fimicolochytrium alabamae MT730755
Fimicolochytrium alabamae MT730939
Fimicolochytrium jonesii MT730754
Fimicolochytrium jonesii MT730626
* Phlyctochytrium arcticum MT730762
* Phlyctochytrium arcticum MT730764
Spizellomyces lactosolyticus MF576127
Gallinipes pseudodichotomus GQ499378
Gallinipes pseudodichotomus AF216764
Gallinipes pseudodichotomus GQ160458
Gallinipes pseudodichotomus MT730752
Gallinipes pseudodichotomus MT730697
Spizellomyces pseudodichotomus MT730719
Spizellomyces plurigibbosus AF216762
Spizellomyces plurigibbosus NR_166002
Spizellomyces plurigibbosus MH860788
Spizellomyces plurigibbosus AF216768
Spizellomyces dolichospermus AF216771
Spizellomyces acuminatus MT730968
Spizellomyces kniepii AF216769
Spizellomyces acuminatus AF216757
Gaertneriomyces semiglobifer MF576124
Gaertneriomyces semiglobifer MF576126
Gaertneriomyces semiglobifer MF576122
Gaertneriomyces semiglobifer MF576117
Gaertneriomyces semiglobifer MT731022
Gaertneriomyces semiglobifer MT730763
Gaertneriomyces semiglobifer MT730738
Gaertneriomyces semiglobifer MT730616
Gaertneriomyces semiglobifer MF576120
Gaertneriomyces semiglobifer MF576119
Gaertneriomyces semiglobifer MF576121
Gaertneriomyces spectabilis MT730737
Gaertneriomyces spectabilis MT730631
Gaertneriomyces semiglobifer MF576123
Gaertneriomyces semiglobifer MF576125
Gaertneriomyces semiglobifer AF216760
Gaertneriomyces semiglobifer MF576118
Kochiomyces dichotomus ON963327
Kochiomyces dichotomus AF216758
Kochiomyces dichotomus MT730708
Kochiomyces dichotomus ON963791
Quaeritorhiza haematococci NR_171267
Quaeritorhiza haematococci MT730893
Rhizophlyctis rosea MT730728
Rhizophlyctis rosea MT730808
Rhizophlyctis rosea KR349619
Rhizophlyctis rosea MT596195
Rhizophlyctis rosea MT730928
Rhizophlyctis rosea MT730947
Rhizophlyctis rosea MT730930
Rhizophlyctis rosea MT595558
Rhizophlyctis rosea MT730933
Rhizophlyctis rosea AF216765
Paranamyces uniporus MT730781
Paranamyces uniporus MT731013
Paranamyces uniporus MT731025
Paranamyces uniporus MT731010
Paludomyces mangrovei NR_138404
Paludomyces mangrovei KP723822
Halomyces littoreus DQ485604
Dinomyces arenysensis KJ027546
Dinomyces arenysensis KJ027548
Dinomyces arenysensis KJ027544
Dinomyces arenysensis KJ027542
Dinomyces arenysensis KJ027540
Dinomyces gilberthii OQ865234
Aquamyces chlorogonii JN943815
Aquamyces chlorogonii DQ485600
Aquamyces chlorogonii NR_119645
Aquamyces chlorogonii EF585643
Angulomyces ubonensis OR051767
Angulomyces ubonensis OR051768
Angulomyces argentinensis MT731008
Protrudomyces lateralis NR_119650
Protrudomyces lateralis EF585668
Protrudomyces lateralis EF585669
Protrudomyces lateralis EF585658
Rhizophydium carpophilum DQ485612
Protrudomyces lateralis DQ485597
Ulkenomyces aestuari DQ485605
Ulkenomyces aestuari DQ485676
Globomyces pollinispini EF585665
Globomyces pollinispini EF585666
Globomyces pollinispini NR_119649
Globomyces pollinispini MT731005
Globomyces pollinispini MT730693
Globomyces pollinispini DQ485596
Globomyces pollinispini MT731004
Urceomyces sphaerocarpus EF585659
Urceomyces sphaerocarpus MT730623
Urceomyces sphaerocarpus NR_119648
Alphamyces chaetifer EF585633
Alphamyces chaetifer EF585663
Pateramyces pingflumenensis OR051766
Rhizophydium globosum MT730690
Rhizophydium brooksianum MT731000
Rhizophydium globosum DQ485616
Rhizophydium globosum MH863084
Operculomyces laminatus NR_119590
Operculomyces laminatus DQ485617
Operculomyces laminatus MT730691
Terramyces subangulosum DQ485673
Terramyces subangulosum NR_119592
Terramyces subangulosum DQ485653
Terramyces subangulosum DQ485652
Boothiomyces macroporosus MT731017
Terramyces aquatica OR051774
Rhizophydium sphaerotheca KJ464412
Clydaea vesicula MT730721
Clydaeav esicula EU352773
Clydaea vesicula NR_121339
Lobulomyces poculatus EU352770
Lobulomyces poculatus EU352771
Lobulomyces poculatus NR_158461
Lobulomyces poculatus MT595869
Joblinomyces apicalis MT085672
Joblinomyces apicalis MT085673
Joblinomyces apicalis MT085671
Joblinomyces apicalis MT085670
Joblinomyces apicalis MT085669
Joblinomyces apicalis MT085674
Anaeromyces robustus KY399731
Anaeromyces robustus NR_148182
Anaeromyces robustus OM238188
Neocallimastix cameroonii MT085722
Piromyces communis MW341535
Piromyces communis MK775325
Joblinomyces apicalis MT085665
Piromyces finnis OL957166
Piromyces finnis NR_148181
Piromyces finnis KY399730
Anaeromyces mucronatus MW899535
Oontomyces anksri ON514405
0.2

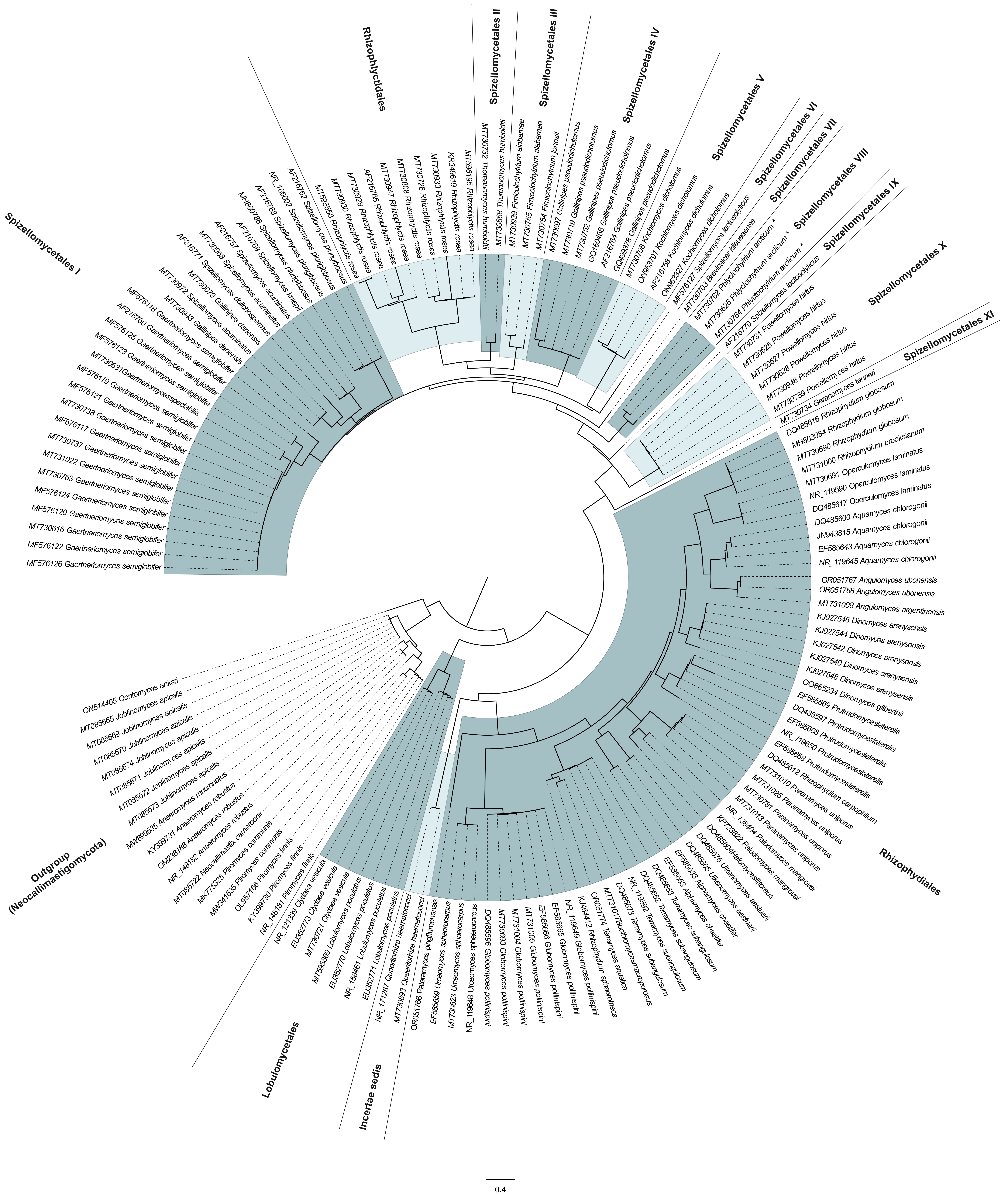
Rhizophlyctidales
Spizellomycetales II
Spizellomycetales III
Spizellomycetales IV
Spizellomycetales V
Spizellomycetales VI
Spizellomycetales VII
Spizellomycetales VIII
Spizellomycetales IX
Spizellomycetales X
Spizellomycetales XI
Spizellomycetales I
Rhizophydiales
Lobulomycetales
Incertae sedis
Outgroup (Neocallimastigomycota)
MF576126 Gaertneriomyces semiglobifer
MF576122 Gaertneriomyces semiglobifer
MT730616 Gaertneriomyces semiglobifer
MF576120 Gaertneriomyces semiglobifer
MF576124 Gaertneriomyces semiglobifer
MT730763 Gaertneriomyces semiglobifer
MT731022 Gaertneriomyces semiglobifer
MT730737 Gaertneriomyces semiglobifer
MF576117 Gaertneriomyces semiglobifer
MT730738 Gaertneriomyces semiglobifer
MF576121 Gaertneriomyces semiglobifer
MF576119 Gaertneriomyces semiglobifer
MT730631Gaertneriomycesspectabilis
MF576123 Gaertneriomyces semiglobifer
MF576125 Gaertneriomyces semiglobifer
AF216760 Gaertneriomyces semiglobifer
MF576118 Gaertneriomyces semiglobifer
MT730943 Gallinipes danensis
MT730972 Spizellomyces acuminatus
MT730679 Gallinipes danensis
AF216771 Spizellomyces dolichospermus
MT730968 Spizellomyces acuminatus
AF216757 Spizellomyces kniepii
AF216769 Spizellomyces acuminatus
MH860788 Spizellomyces plurigibbosus
AF216768 Spizellomyces plurigibbosus
NR_166002 Spizellomyces plurigibbosus
AF216762 Spizellomyces plurigibbosus
MT595558 Rhizophlyctis rosea
MT730930 Rhizophlyctis rosea
MT730928 Rhizophlyctis rosea
AF216765 Rhizophlyctis rosea
MT730947 Rhizophlyctis rosea
MT730808 Rhizophlyctis rosea
MT730728 Rhizophlyctis rosea
MT730933 Rhizophlyctis rosea
KR349619 Rhizophlyctis rosea
MT596195 Rhizophlyctis rosea
MT730732 Thoreauomyces humboldtii
MT730668 Thoreauomyces humboldtii
MT730939 Fimicolochytrium alabamae
MT730755 Fimicolochytrium alabamae
MT730754 Fimicolochytrium jonesii
MT730697 Gallinipes pseudodichotomus
MT730719 Gallinipes pseudodichotomus
MT730752 Gallinipes pseudodichotomus
GQ160458 Gallinipes pseudodichotomus
AF216764 Gallinipes pseudodichotomus
GQ499378 Gallinipes pseudodichotomus
MT730708 Kochiomyces dichotomus
ON963791 Kochiomyces dichotomus
AF216758 Kochiomyces dichotomus
ON963327 Kochiomyces dichotomus
MF576127 Spizellomyces lactosolyticus
MT730703 Brevicalcar kilaueaense
MT730762 Phlyctochytrium arcticum *
MT730626 Phlyctochytrium arcticum *
MT730764 Phlyctochytrium arcticum *
AF216770 Spizellomyces lactosolyticus
MT730731 Powellomyces hirtus
MT730625 Powellomyces hirtus
MT730627 Powellomyces hirtus
MT730628 Powellomyces hirtus
MT730946 Powellomyces hirtus
MT730759 Powellomyces hirtus
MT730734 Geranomyces tanneri
DQ485616 Rhizophydium globosum
MH863084 Rhizophydium globosum
MT730690 Rhizophydium globosum
MT731000 Rhizophydium brooksianum
MT730691 Operculomyces laminatus
NR_119590 Operculomyces laminatus
DQ485617 Operculomyces laminatus
DQ485600 Aquamyces chlorogonii
JN943815 Aquamyces chlorogonii
EF585643 Aquamyces chlorogonii
NR_119645 Aquamyces chlorogonii
OR051767 Angulomyces ubonensis
OR051768 Angulomyces ubonensis
MT731008 Angulomyces argentinensis
KJ027546 Dinomyces arenysensis
KJ027544 Dinomyces arenysensis
KJ027542 Dinomyces arenysensis
KJ027540 Dinomyces arenysensis
KJ027548 Dinomyces arenysensis
OQ865234 Dinomyces gilberthii
EF585669 Protrudomyceslateralis
DQ485597 Protrudomyceslateralis
EF585668 Protrudomyceslateralis
NR_119650 Protrudomyceslateralis
EF585658 Protrudomyceslateralis
DQ485612 Rhizophydium carpophilum
MT731010 Paranamyces uniporus
MT731025 Paranamyces uniporus
MT730781 Paranamyces uniporus
MT731013 Paranamyces uniporus
NR_138404 Paludomyces mangrovei
KP723822 Paludomyces mangrovei
DQ485604Halomyceslittoreus
DQ485605 Ulkenomyces aestuarii
DQ485676 Ulkenomyces aestuarii
EF585633 Alphamyces chaetifer
EF585663 Alphamyces chaetifer
DQ485653 Terramyces subangulosum
DQ485652 Terramyces subangulosum
NR_119592 Terramyces subangulosum
DQ485673 Terramyces subangulosum
MT731017Boothiomycesmacroporosus
OR051774 Terramyces aquatica
KJ464412 Rhizophydium sphaerotheca
NR_119649 Globomyces pollinispini
EF585665 Globomyces pollinispini
EF585666 Globomyces pollinispini
MT731005 Globomyces pollinispini
MT731004 Globomyces pollinispini
MT730693 Globomyces pollinispini
DQ485596 Globomyces pollinispini
NR_119648 Urceomyces sphaerocarpus
MT730623 Urceomyces sphaerocarpus
EF585659 Urceomyces sphaerocarpus
OR051766 Pateramyces pingflumenensis
MT730893 Quaeritorhiza haematococci
NR_171267 Quaeritorhiza haematococci
EU352771 Lobulomyces poculatus
NR_158461 Lobulomyces poculatus
EU352770 Lobulomyces poculatus
MT595869 Lobulomyces poculatus
MT730721 Clydaea vesicula
EU352773 Clydaea vesicula
NR_121339 Clydaea vesicula
NR_148181 Piromyces finnis
KY399730 Piromyces finnis
OL957166 Piromyces finnis
MW341535 Piromyces communis
MK775325 Piromyces communis
MT085722 Neocallimastix cameroonii
NR_148182 Anaeromyces robustus
OM238188 Anaeromyces robustus
KY399731 Anaeromyces robustus
MW899535 Anaeromyces mucronatus
MT085673 Joblinomyces apicalis
MT085672 Joblinomyces apicalis
MT085671 Joblinomyces apicalis
MT085674 Joblinomyces apicalis
MT085670 Joblinomyces apicalis
MT085669 Joblinomyces apicalis
MT085665 Joblinomyces apicalis
ON514405 Oontomyces anksri
0.4

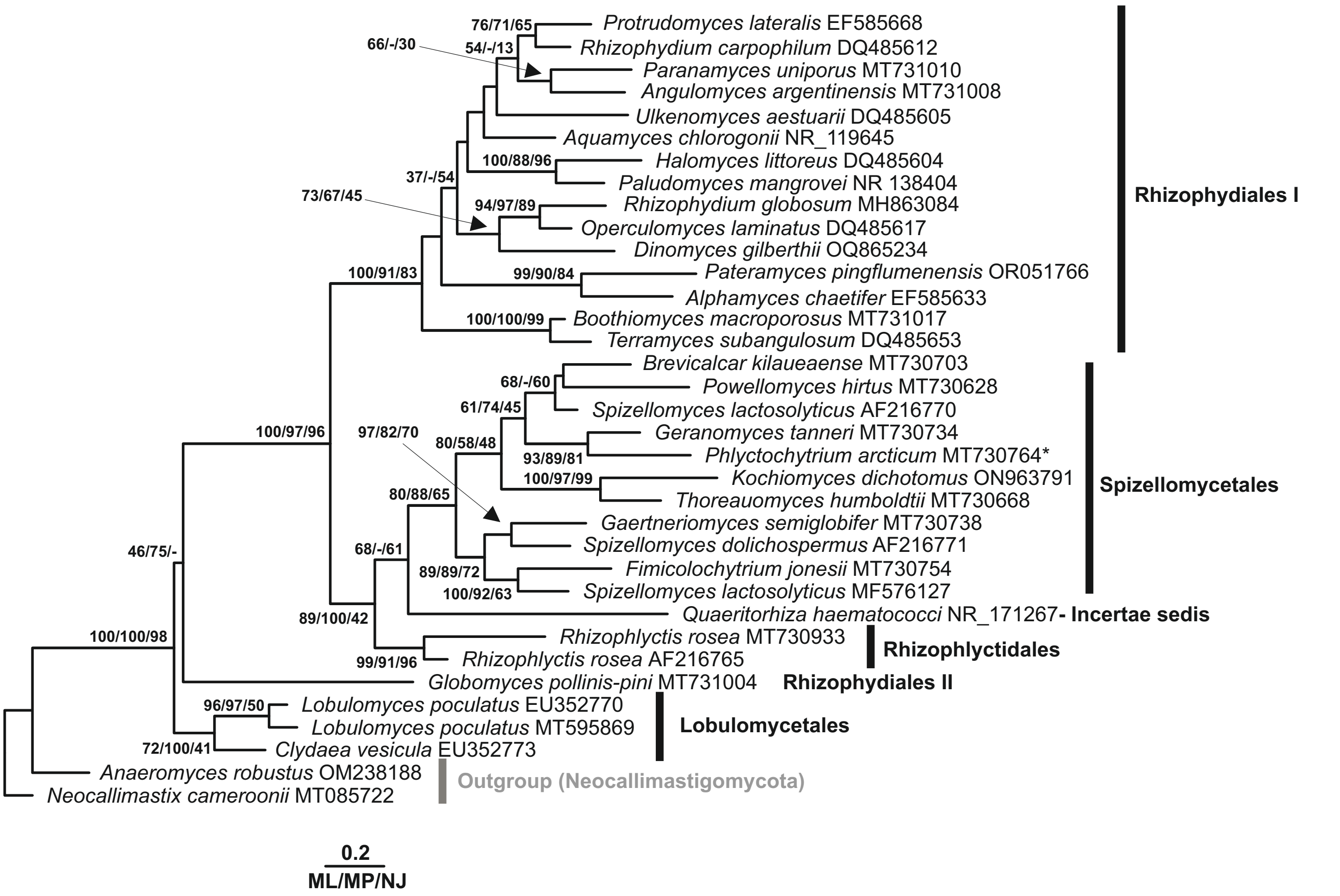

Protrudomyces lateralis EF585668
Rhizophydium carpophilum DQ485612
Paranamyces uniporus MT731010
Angulomyces argentinensis MT731008
Ulkenomyces aestuarii DQ485605
Aquamyces chlorogonii NR_119645
Halomyces littoreus DQ485604
Paludomyces mangrovei NR 138404
Rhizophydium globosum MH863084
Operculomyces laminatus DQ485617
Dinomyces gilberthii OQ865234
Pateramyces pingflumenensis OR051766
Alphamyces chaetifer EF585633
Boothiomyces macroporosus MT731017
Terramyces subangulosum DQ485653
Brevicalcar kilaueaense MT730703
Powellomyces hirtus MT730628
Spizellomyces lactosolyticus AF216770
Geranomyces tanneri MT730734
Phlyctochytrium arcticum MT730764*
Kochiomyces dichotomus ON963791
Thoreauomyces humboldtii MT730668
Gaertneriomyces semiglobifer MT730738
Spizellomyces dolichospermus AF216771
Fimicolochytrium jonesii MT730754
Spizellomyces lactosolyticus MF576127
Quaeritorhiza haematococci NR_171267- Incertae sedis
Rhizophlyctis rosea MT730933
Rhizophlyctis rosea AF216765
Globomyces pollinis-pini MT731004
Lobulomyces poculatus EU352770
Lobulomyces poculatus MT595869
Clydaea vesicula EU352773
Anaeromyces robustus OM238188
Neocallimastix cameroonii MT085722
Rhizophydiales I
Spizellomycetales
Rhizophlyctidales
Rhizophydiales II
Lobulomycetales
Outgroup (Neocallimastigomycota)
76/71/65
54/-/13
66/-/30
100/88/96
37/-/54
73/67/45
94/97/89
100/91/83
99/90/84
100/100/99
68/-/60
61/74/45
100/97/96
97/82/70
80/58/48
93/89/81
100/97/99
80/88/65
46/75/-
68/-/61
89/89/72
100/92/63
89/100/42
100/100/98
99/91/96
96/97/50
72/100/41
0.2
ML/MP/NJ

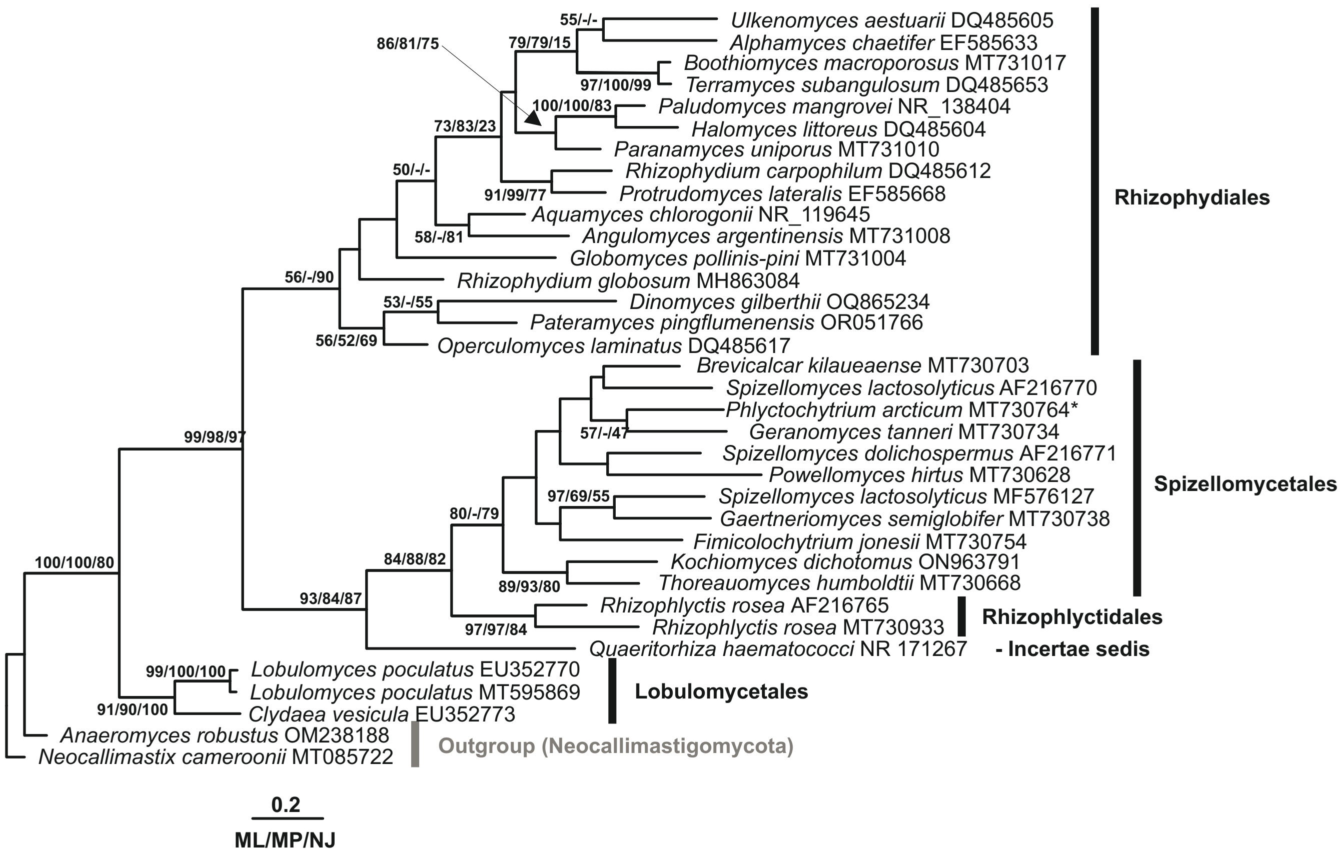
Ulkenomyces aestuarii DQ485605
Alphamyces chaetifer EF585633
Boothiomyces macroporosus MT731017
Terramyces subangulosum DQ485653
Paludomyces mangrovei NR_138404
Halomyces littoreus DQ485604
Paranamyces uniporus MT731010
Rhizophydium carpophilum DQ485612
Protrudomyces lateralis EF585668
Aquamyces chlorogonii NR_119645
Angulomyces argentinensis MT731008
Globomyces pollinis-pini MT731004
Rhizophydium globosum MH863084
Dinomyces gilberthii OQ865234
Pateramyces pingflumenensis OR051766
Operculomyces laminatus DQ485617
Brevicalcar kilaueaense MT730703
Spizellomyces lactosolyticus AF216770
Phlyctochytrium arcticum MT730764*
Geranomyces tanneri MT730734
Spizellomyces dolichospermus AF216771
Powellomyces hirtus MT730628
Spizellomyces lactosolyticus MF576127
Gaertneriomyces semiglobifer MT730738
Fimicolochytrium jonesii MT730754
Kochiomyces dichotomus ON963791
Thoreauomyces humboldtii MT730668
Rhizophlyctis rosea AF216765
Rhizophlyctis rosea MT730933
Quaeritorhiza haematococci NR 171267
Lobulomyces poculatus EU352770
Lobulomyces poculatus MT595869
Clydaea vesicula EU352773
Anaeromyces robustus OM238188
Neocallimastix cameroonii MT085722
Rhizophydiales
Spizellomycetales
Rhizophlyctidales
- Incertae sedis
Lobulomycetales
Outgroup (Neocallimastigomycota)
55/-/-
79/79/15
86/81/75
97/100/99
100/100/83
73/83/23
50/-/-
91/99/77
58/-/81
56/-/90
53/-/55
56/52/69
57/-/47
97/69/55
80/-/79
84/88/82
89/93/80
99/98/97
100/100/80
93/84/87
97/97/84
99/100/100
91/90/100
0.2
ML/MP/NJ

Table S1a. List of all ITS2 rRNA gene templates used in this study. The ITS2 templates in this were obtained from the ITS2 database. All sequences designated as "uncultured" and ".sp" were manually filtered out. The remaining 23 sequences, which included their respective structures, were used as templates for the homology modeling.

Index	GenBank Acc. No.	Organism name	Order
**1.**	AF216764	*Spizellomyces pseudodichotomus*	Spizellomycetales
**2.**	GQ160458	*Spizellomyces pseudodichotomus*	Spizellomycetales
**3.**	GQ499378	*Spizellomyces pseudodichotomus*	Spizellomycetales
**4.**	KJ027542	*Dinomyces arenysensis*	Rhizophydiales
**5.**	KJ027544	*Dinomyces arenysensis*	Rhizophydiales
**6.**	KJ027546	*Dinomyces arenysensis*	Rhizophydiales
**7.**	KJ027548	*Dinomyces arenysensis*	Rhizophydiales
**8.**	AF216760	*Gaertneriomyces semiglobifer*	Spizellomycetales
**9.**	DQ485605	*Rhizophydium aestuarii*	Rhizophydiales
**10.**	DQ485676	*Rhizophydium aestuarii*	Rhizophydiales
**11.**	DQ485617	*Operculomyces laminatus*	Rhizophydiales
**12.**	AF216770	*Spizellomyces lactosolyticus*	Spizellomycetales
**13.**	AF216757	*Spizellomyces acuminatus*	Spizellomycetales
**14.**	AF216765	*Rhizophlyctis rosea*	Rhizophlyctidales
**15.**	AF216771	*Spizellomyces dolichospermus*	Spizellomycetales
**16.**	AF216762	*Spizellomyces plurigibbosus*	Spizellomycetales
**17.**	DQ485612	*Rhizophydium carpophilum*	Rhizophydiales
**18.**	EU352773	*Clydaea vesicula*	Lobomycetales
**19.**	KJ027540	*Dinomyces arenysensis*	Rhizophydiales
**20.**	AF216768	*Spizellomyces plurigibbosus*	Spizellomycetales
**21.**	JN943815	*Aquamyces chlorogonii*	Rhizophydiales
**22.**	NR_119645	*Aquamyces chlorogonii*	Rhizophydiales
**23.**	NR_121339	*Clydaea vesicula*	Lobomycetales

Table S1b. List of all ITS2 rRNA gene sequences used in this study. The following list presents the organisms' names according to NCBI, the GenBank accession number, and the average transfer helix from the homology modeling in %. The templates used for the homology modeling are displayed in Table S1a. To obtain this set of sequences, the uncultured, ".sp", and cloned sequence data were excluded, and a targeted search for "internal transcribed spacer 2" and "ITS2" was conducted. Search string: (Chytridiomycota [Organism] AND "internal transcribed spacer 2" NOT uncultured NOT ".sp" NOT clone) or (Chytridiomycota [Organism] AND "ITS2" NOT uncultured NOT ".sp" NOT clone). Sequences with less than 50% structural homology were discarded.

GenBank Acc. No.	Organism name	Ø Transfer helix [%]
NR_158461	*Lobulomyces poculatus*	60.01675
NR_148181	*Piromyces finnis*	57.06075
OR051774	*Terramyces aquatica*	71.43575
NR_119592	*Terramyces subangulosum*	57.5845
NR_148182	*Anaeromyces robustus*	50.54275
NR_119645	*Aquamyces chlorogonii*	100
NR_119648	*Urceomyces sphaerocarpus*	68.23825
NR_119649	*Globomyces pollinis-pini*	51.6905
NR_119650	*Protrudomyces lateralis*	78.83525
NR_138404	*Paludomyces mangrovei*	57.47
NR_166002	*Spizellomyces plurigibbosus*	100
NR_171267	*Quaeritorhiza haematococci*	51.89675
AF216765	*Rhizophlyctis rosea*	100
AF216762	*Spizellomyces plurigibbosus*	100
AF216760	*Gaertneriomyces semiglobifer*	100
AF216758	*Kochiomyces dichotomus*	51.25
AF216757	*Spizellomyces acuminatus*	100
AF216764	*Gallinipes pseudodichotomus*	100
AF216771	*Spizellomyces dolichospermus*	100
AF216770	*Spizellomyces lactosolyticus*	98.33325
AF216769	*Spizellomyces kniepii*	83.96825
AF216768	*Spizellomyces plurigibbosus*	100

EU352771	*Lobulomyces poculatus*	60.01675
EU352770	*Lobulomyces poculatus*	65.54425
GQ499378	*Gallinipes pseudodichotomus*	100
GQ160458	*Gallinipes pseudodichotomus*	100
JN943815	*Aquamyces chlorogonii*	100
OR051768	*Angulomyces ubonensis*	81.80075
OR051767	*Angulomyces ubonensis*	81.80075
OR051766	*Pateramyces pingflumenensis*	61.84525
OQ865234	*Dinomyces gilberthii*	69.696
MH863084	*Rhizophydium globosum*	69.13275
MH860788	*Spizellomyces plurigibbosus*	100
ON514405	*Oontomyces anksri*	56.30075
ON963791	*Kochiomyces dichotomus*	53.0555
ON963327	*Kochiomyces dichotomus*	62.8885
OM238188	*Anaeromyces robustus*	50.54275
OL957166	*Piromyces finnis*	56.38525
MW899535	*Anaeromyces mucronatus*	50.827
MW341535	*Piromyces communis*	58.013
MT731025	*Paranamyces uniporus*	77.2635
MT731022	*Gaertneriomyces semiglobifer*	91.541
MT731017	*Boothiomyces macroporosus*	70.96275
MT731013	*Paranamyces uniporus*	77.93925
MT731010	*Paranamyces uniporus*	77.147
MT731008	*Angulomyces argentinensis*	78.8515
MT731004	*Globomyces pollinis-pini*	53.7885
MT731000	*Rhizophydium brooksianum*	68.497
MT731005	*Globomyces pollinis-pini*	51.6905
MT730972	*Spizellomyces acuminatus*	80.16725
MT730968	*Spizellomyces acuminatus*	88.9285
MT730947	*Rhizophlyctis rosea*	74.17075
MT730943	*Gallinipes danensis*	80.16725
MT730928	*Rhizophlyctis rosea*	77.11925
MT730933	*Rhizophlyctis rosea*	60.60325
MT730939	*Fimicolochytrium alabamae*	55.27775
MT730930	*Rhizophlyctis rosea*	61.88575
MT730893	*Quaeritorhiza haematococci*	51.89675
MT730808	*Rhizophlyctis rosea*	76.68925
MT730781	*Paranamyces uniporus*	77.93925
MT730946	*Powellomyces hirtus*	56.386
MT730764	*Phlyctochytrium arcticum*	54.6815
MT730763	*Gaertneriomyces semiglobifer*	91.541
MT730762	*Phlyctochytrium arcticum*	51.6665
MT730759	*Powellomyces hirtus*	56.386
MT730755	*Fimicolochytrium alabamae*	55.27775
MT730754	*Fimicolochytrium jonesii*	67.91675
MT730752	*Gallinipes pseudodichotomus*	100
MT730738	*Gaertneriomyces semiglobifer*	91.541
MT730737	*Gaertneriomyces semiglobifer*	89.335
MT730679	*Gallinipes danensis*	80.16725
MT730690	*Rhizophydium globosum*	68.497
MT730691	*Operculomyces laminatus*	100
MT730693	*Globomyces pollinis-pini*	51.6905
MT730697	*Gallinipes pseudodichotomus*	65.6945
MT730703	*Brevicalcar kilaueaense*	72.8865
MT730708	*Kochiomyces dichotomus*	59.5835
MT730719	*Gallinipes pseudodichotomus*	100
MT730721	*Clydaea vesicula*	94.27475
MT730728	*Rhizophlyctis rosea*	75.092
MT730731	*Powellomyces hirtus*	56.386
MT730732	*Thoreauomyces humboldtii*	72.3895
MT730734	*Geranomyces tanneri*	62.55075
MT730668	*Thoreauomyces humboldtii*	70.2635
MT730631	*Gaertneriomyces spectabilis*	90.389
MT085674	*Joblinomyces apicalis*	50.64675
MT730628	*Powellomyces hirtus*	56.386
MT730627	*Powellomyces hirtus*	57.99875
MT730626	*Phlyctochytrium arcticum*	51.6665
MT730625	*Powellomyces hirtus*	56.386

MT730623	*Urceomyces sphaerocarpus*	68.23825
MT730616	*Gaertneriomyces semiglobifer*	91.541
MT596195	*Rhizophlyctis rosea*	76.0135
MT595869	*Lobulomyces poculatus*	58.1755
MT595558	*Rhizophlyctis rosea*	61.88575
MT085722	*Neocallimastix cameroonii*	53.978
MT085673	*Joblinomyces apicalis*	50.64675
MT085672	*Joblinomyces apicalis*	50.64675
MT085671	*Joblinomyces apicalis*	50.64675
MT085670	*Joblinomyces apicalis*	50.64675
MT085669	*Joblinomyces apicalis*	50.64675
MT085665	*Joblinomyces apicalis*	55.46475
DQ485676	*Ulkenomyces aestuarii*	100
DQ485673	*Terramyces subangulosum*	57.5845
DQ485653	*Terramyces subangulosum*	57.5845
DQ485652	*Terramyces subangulosum*	57.5845
DQ485616	*Rhizophydium globosum*	67.976
DQ485612	*Rhizophydium carpophilum*	100
DQ485605	*Ulkenomyces aestuarii*	100
DQ485604	*Halomyces littoreus*	70.017
DQ485600	*Aquamyces chlorogonii*	100
DQ485597	*Protrudomyces lateralis*	68.52675
DQ485596	*Globomyces pollinis-pini*	51.6905
KP723822	*Paludomyces mangrovei*	57.47
EF585669	*Protrudomyces lateralis*	78.83525
EF585668	*Protrudomyces lateralis*	78.83525
EF585666	*Globomyces pollinis-pini*	51.6905
EF585665	*Globomyces pollinis-pini*	51.6905
EF585663	*Alphamyces chaetifer*	58.2095
EF585659	*Urceomyces sphaerocarpus*	68.23825
EF585658	*Protrudomyces lateralis*	74.14775
EF585643	*Aquamyces chlorogonii*	100
EF585633	*Alphamyces chaetifer*	58.2095
MK775325	*Piromyces communis*	54.83975
MF576127	*Spizellomyces lactosolyticus*	78.61125
MF576126	*Gaertneriomyces semiglobifer*	91.541
MF576125	*Gaertneriomyces semiglobifer*	100
MF576124	*Gaertneriomyces semiglobifer*	91.541
MF576123	*Gaertneriomyces semiglobifer*	89.65375
MF576122	*Gaertneriomyces semiglobifer*	91.541
MF576121	*Gaertneriomyces semiglobifer*	91.541
MF576120	*Gaertneriomyces semiglobifer*	91.541
MF576119	*Gaertneriomyces semiglobifer*	91.541
MF576118	*Gaertneriomyces semiglobifer*	81.9615
MF576117	*Gaertneriomyces semiglobifer*	91.541
KY399730	*Piromyces finnis*	57.06075
KY399731	*Anaeromyces robustus*	50.54275
KR349619	*Rhizophlyctis rosea*	76.0135
KJ464412	*Rhizophydium sphaerotheca*	65.5575
KJ027548	*Dinomyces arenysensis*	100
KJ027546	*Dinomyces arenysensis*	100
KJ027544	*Dinomyces arenysensis*	100
KJ027542	*Dinomyces arenysensis*	100
KJ027540	*Dinomyces arenysensis*	100
DQ485617	*Operculomyces laminatus*	100
EU352773	*Clydaea vesicula*	100
NR_121339	*Clydaea vesicula*	100
NR_119590	*Operculomyces laminatus*	100
